# Opioid Use at End-Of-Life Among Nova Scotia Patients With Cancer

**DOI:** 10.3389/fphar.2022.836864

**Published:** 2022-03-24

**Authors:** Laura V. Minard, Judith Fisher, Larry Broadfield, Gordon Walsh, Ingrid Sketris

**Affiliations:** ^1^ College of Pharmacy, Dalhousie University, Halifax, NS, Canada; ^2^ Nova Scotia Department of Health and Wellness, Halifax, NS, Canada; ^3^ Nova Scotia Health Cancer Care Program, Halifax, NS, Canada

**Keywords:** palliative, pain control, pharmacy, opioids, cancer, end-of-life (EOL), linked data (data linkage), oncology

## Abstract

**Purpose:** To determine the factors associated with opioid analgesic prescriptions as measured by community pharmacy dispensations to all Nova Scotia (NS) patients with cancer at end-of-life from 2005 to 2009.

**Methods:** The NS Cancer Registry and the NS Prescription Monitoring Program (NSPMP) were used to link Nova Scotians who had a cancer diagnosis and received a prescription for opioids in their last year of life (*n* = 6,186) from 2005 to 2009. The association of factors with opioid dispensations at end-of-life were determined (e.g., patient demographics, type of prescriber, type of cancer, and opioid type, formulation, and dose).

**Results:** Almost 54% (*n* = 6,186) of the end-of-life study population with cancer (*n* = 11,498) was linked to the NSPMP and therefore dispensed opioids. Most prescriptions were written by general practitioners (89%) and were for strong opioids (81%). Immediate-release formulations were more common than modified-release formulations. Although the annual average parenteral morphine equivalents (MEQ) did not change during the study period, the number of opioid prescriptions per patient per year increased from 5.9 in 2006 to 7.0 in 2009 (*p* < 0.0001). Patients age 80 and over received the fewest prescriptions (mean 3.9/year) and the lowest opioid doses (17.0 MEQ) while patients aged 40–49 received the most prescriptions (mean 14.5/year) and the highest doses of opioid (80.2 MEQ).

**Conclusion:** Our study examined opioid analgesic use at end-of-life in patients with cancer for a large real-world population and determined factors, trends and patterns associated with type and dose of opioid dispensed. We provide information regarding how general practitioners prescribe opioid therapy to patients at end-of-life. Our data suggest that at the time of this study, there may have been under-prescribing of opioids to patients with cancer at end-of-life. This information can be used to increase awareness among general practitioners, and to inform recommendations from professional regulatory bodies, to aid in managing pain for cancer patients at end-of-life. Future work could address how opioid prescribing has changed over time, and whether efforts to reduce opioid prescribing in response to the opioid crisis have affected patients with cancer at end-of-life in Nova Scotia.

## Introduction

Pain is common among persons with cancer. A meta-analysis of 52 studies found that 64% of patients with metastatic or advanced cancer, and 59% of patients in active cancer treatment experienced pain, and more than one-third of patients with pain characterized their pain as moderate or severe (van den Beuken-van Everdingen et al., 2007). Other literature reports that 80–90% of patients with metastatic cancer experience pain, primarily due to tumour infiltration (Christo and Mazloomdoost, 2008; Jost et al., 2010). Cancer survivors may also experience chronic pain that is related to their treatment, such as surgery, chemotherapy or radiation, tissue damage from the malignancy and/or cancer-related conditions (Levy et al., 2008). For example, the incidence of post-surgical chronic pain among breast cancer survivors is estimated to be as high as 50% (Burton et al., 2007).

Strategies exist that can effectively manage cancer-related pain. The World Health Organization (WHO) guidelines for cancer pain management, the “3-step analgesic ladder,” position opioids at the second and third steps of the ladder (World Health Organization, 1996). Step 2, for moderate pain, includes weak, immediate-release opioids such as codeine or tramadol, possibly in combination with the non-opioid analgesic, acetaminophen, or a non-steroidal anti-inflammatory drug (Hanks et al., 2001; Krakowski et al., 2003). Strong opioids are recommended for moderate to severe pain (step 3) (World Health Organization, 1996). More recently, many guideline groups have proposed alterations to the WHO analgesic ladder, including deleting the second step, and recommending early use of low dose morphine (Ripamonti et al., 2011; Bandieri et al., 2016; Fallon, 2017; Pain and symptom management, 2017). In addition, there are concerns about using tramadol given its dual mechanism of action, unpredictable metabolism, potential for withdrawal, and toxicities (Young and Juurlink, 2013; Nelson and Juurlink, 2015; Morrow et al., 2019).

Opioid analgesics offer an overall favorable risk to benefit profile (Christo and Mazloomdoost, 2008) and are the mainstay of the pharmacological management of moderate to severe cancer-related pain (Hanks et al., 2001; Krakowski et al., 2003; Henderson, 2017; Pain and symptom management, 2017; Wiffen et al., 2017). A 2017 Cochrane review concluded that with opioid use, approximately 95% of patients with cancer could have their pain reduced from moderate or severe to mild or no pain within 14 days (Wiffen et al., 2017).

Morphine is a strong (step 3) opioid of first choice and the standard against which other opioid analgesics are measured (Hanks et al., 2001). However, patients vary in their response to opioids and some patients may benefit from the use of alternative strong opioids including hydromorphone, oxycodone, fentanyl, and methadone (Breivik, 2001; Hanks et al., 2001). For the majority of patients, the preferred route of administration is oral (Krakowski et al., 2003); however, transdermal, subcutaneous, intramuscular or intravenous routes (occasionally) may be necessary for patients who are unable to take oral medications.

Expert opinion estimates that adequate pain control is possible for 90% of patients with cancer (Cleary, 2007; Deandrea et al., 2008). However, the undertreatment of cancer-related pain is common and a substantial percentage of patients with cancer experience inadequate pain control (Cleary, 2007; Christo and Mazloomdoost, 2008; Deandrea et al., 2008). Barriers to adequate pain management among cancer patients can arise from patient, prescriber, and system level factors (Christo and Mazloomdoost, 2008; Deandrea et al., 2008). The purpose of this study was to 1) determine the use of opioids in Nova Scotia patients with cancer at end-of-life, 2) assess the factors, trends and patterns associated with opioid analgesic prescriptions, and 3) measure opioid dispensing over time from 2005 to 2009.

## Materials and Methods

This study was approved by the Capital District Health Authority Research Ethics Board, the Nova Scotia Department of Health and Wellness, the Nova Scotia Prescription Monitoring Program Board, and the Cancer Care Nova Scotia Research Committee. Data were de-identified and analyzed after the patient population was deceased; therefore, informed consent was not obtained.

### Data Sources

Two data sources used were the Nova Scotia Prescription Monitoring Program database (Nova Scotia Prescription Monitoring Program, 2017) and the Nova Scotia Cancer Registry database (International Association of Cancer Registries, 2018).

#### The Nova Scotia Prescription Monitoring Program

The Nova Scotia Prescription Monitoring Program (NSPMP) database is an electronic database maintained by Medavie Blue Cross, the organization that administers the province’s health insurance program on behalf of the NS government (Nova Scotia Prescription Monitoring Program, 2017). With few exceptions (e.g., products containing tramadol), opioid prescriptions are required by provincial legislation to be reported to the NSPMP. Therefore, the electronic database contains data on most prescription opioid analgesics dispensed by community pharmacies in NS since 1 July 2005. The NSPMP includes comprehensive drug, patient and prescriber related data such as drug name, type, dosage form, quantity dispensed, days supply, patient sex, patient birthdate, and prescriber type (Fisher et al., 2012; Furlan et al., 2014; Nova Scotia Prescription Monitoring Program, 2017). Reporting is completed by the community pharmacy at the time that the prescription is received. Opioid prescriptions for patients residing in long-term care are supplied by community pharmacies and included in the NSPMP while prescriptions for patients admitted to hospital and the limited number of patients who reside in long-term care within the hospital system are supplied by the hospital pharmacy and are not reported to the NSPMP.

#### The Nova Scotia Cancer Registry

Cancer is a reportable disease in NS. The Nova Scotia Cancer Registry (NSCR) has been collecting data on cancers diagnosed in the province since 1964 (International Association of Cancer Registries, 2018). The registry excludes non-melanoma skin cancers. The registry contains patient demographics as well as cancer characteristics, such as prognostic information. The International Classification of Diseases for Oncology is used as the standard classification system to define and categorize each new case within the NSCR. Additional reporting guidelines are set out by the Canadian Cancer Registry at Statistics Canada. Each cancer is counted only once, at the time it is diagnosed. This means that if a patient’s cancer goes into remission or if the cancer is considered to be under control, but symptoms reappear at a later date it is not counted again. The cause of death is obtained from the death certificate that is provided to the NSCR from the Vital Statistics Unit of Service Nova Scotia, Government of Nova Scotia.

### Data Linkage

Processes and procedures to preserve the privacy and confidentiality for the sensitive data present in both data sets were established (Supplementary Figure S1). A multi-phased approach was utilized whereby the identification of cases to define the study population was separated from the subsequent construction of the analytic data file. The initial data linkage was undertaken using only identifiers necessary for probabilistic record linkage. No data elements such as prescription data or cancer treatment information were included at the data linkage stage. Furthermore, the analysts involved in the record linkage process were not involved in the data analysis. Conversely, the analysts who conducted the data analysis were not involved in the linkage process and had no access to personal identifiers (Fisher et al., 2011; Broadfield et al., 2018a; Broadfield et al., 2018b; Fisher et al., 2018).

### Study Population

The analytical data file included all NS residents diagnosed with cancer from 1991 onward and living in NS during the period 2005–2009. Opioid prescriptions included were those dispensed between 1 July 2005 and 31 December 2009.

Some persons had two or more cancer diagnoses (i.e., two or more cases for one person). In total, there were 53,618 individual persons who had 62,329 tumours (or cases) in the NSCR. The overall linked population consisted of 26,439 cancer cases, representing 25,360 people. For individuals with more than one cancer diagnosis, opioid usage data was assigned only to the most recent case, so 8,711 cases were not assigned any opioid usage by analysis design. Of note, 30,121 cases (or 48% of the total cases) were not linked between the two databases, which indicates that there was no opioid therapy dispensed in community pharmacies for these persons.

The end-of-life study population included those cancer cases that were deceased between 1 July 2006 and 31 December 2009. End-of-life was defined as the last 12 months of life (Victoria State Government and Health and Human Services, 2016). Although we recognize that end-of-life may be shorter or longer than 12 months for the individual patient, we used 12 months to capture a broad range of cancer diagnoses and patients. Opioid prescriptions were restricted to include those dispensed within the 12-month period preceding death. Based on these definitions, 11,498 persons were defined as end-of-life, of which 6,186 (54%) were found in the NSPMP database.

### Calculation of Morphine Equivalent Daily Dose

For each cancer case at end-of-life, prescribed daily doses were calculated (University of Manitoba, 2005). The sum of all opioid prescriptions filled in community pharmacies within the 12 months preceding death was determined. Morphine equivalents per day (MEQ) were calculated because different opioids have different potencies. The MEQ is a calculation used to normalize different opioids to a single standard. The MEQ is expressed in milligrams and reported as parenteral morphine equivalents. The morphine equivalents used for morphine, hydromorphone, codeine, and oxycodone were 1, 5 (Alberta Cancer Board, 2001), 0.05 and 0.334, respectively. For fentanyl, 1 mg of drug was considered equivalent to 300 mg of morphine based on a 3-days supply. For each opioid prescription dispensed, the MEQ were calculated by dividing the dispensed quantity by the days’ supply and multiplying the quotient by the morphine equivalent associated with the opioid in question. For oral solutions, it was assumed that each dosage was 5 ml, and the dispensed quantity was divided by 5 to make them equivalent with tablet/capsule units. Parenteral dosages were inadvertently divided by 5 due to an error in data analysis. These values were summed for the 12 months preceding death and then divided by the total number of days to derive a daily average. The total number of days was estimated based on the presumed duration of the prescription, which was assumed from the dose and quantity prescribed.

Methadone, dextropropoxyphene, meperidine and pentazocine prescriptions were excluded from these calculations; these agents do not have reliable equianalgesic conversion values, so MEQs cannot be calculated.

### Method of Estimation of Chronic Opioid Use

Chronic pain was estimated using duration and amount of opioids. For tablets and oral solutions, chronic use was defined as use of 360 or more tablets/oral agents in a 90-day period. Use of modified-release agents automatically qualified as chronic use.

### Data Analysis

The frequency and proportion of persons with cancer at end-of-life dispensed opioid analgesics and their type, quantity, daily dosage, and route of administration were determined. The drugs studied were: morphine (ATC: N02AA01); oxycodone (ATC: N02AA05); fentanyl (ATC: N02AB03); codeine (ATC: R05DA04); hydromorphone (ATC: N02AA03); acetylsalicylic acid/opioid combinations (ATC: N02BA51); acetaminophen/opioid combinations (ATC: N02BE51, N02AA59); methadone (ATC: N07BC02); buprenorphine (ATC: N02AE01) (excludes buprenorphine combination with naloxone (Suboxone) oral tablets (ATC: N07BC51) which are prescribed for opioid dependency); dextropropoxyphene (ATC: N02AC04); meperidine (ATC: N02AB02); and pentazocine (ATC: N02AD01) (WHO Collaborating Centre for Drug Statistics Methodology, 2009).

The association of the following factors with opioid dispensations at end-of-life was determined:

1) Patient sex,
2) Patient age group at diagnosis by decade (0–29, 30–39,40–49, 50–59, 60–69, 70–79 or 80+). Children and young adults were combined due to the relatively low prevalence of cancers in this group,
3) Patient place of residence by rural/urban designation,
4) Prescriber type: general practitioner or specialist,
5) Cancer site/type was based on the Canadian Cancer Statistics framework (Canadian Cancer Society’s Steering Committee, 2010) and classified as follows: oral, esophagus, stomach, colorectal, pancreas, larynx, lung, skin, breast, cervix, body of uterus, ovary, prostate, testis, bladder, kidney, brain, thyroid, non-Hodgkin’s lymphoma, Hodgkin’s lymphoma, leukemia, liver, multiple myeloma, and other cancers (small bowel, peritoneum and gastrointestinal unspecified, paranasal sinuses, mediastinum, other female genital, penis and male genital unspecified, eye and lacrimal gland, endocrine and other, bone and connective tissue, miscellaneous proliferative disease, other ill defined, unknown primary, and non-melanoma),
6) Prognostic tier was the probability of 5-year survival for each site (Ellison and Wilkins, 2010), with compilation of each site into one of the three groups: high probability of 5-year survival (>80%; tier 1), intermediate probability of 5-year survival (50–80%; tier 2), or low probability of 5-year survival (<50%; tier 3),
7) Opioid formulation: immediate-release (tablets and capsules, powders, suppositories, or oral solutions), modified-release (tablets and capsules, transdermal patches or discs) or miscellaneous, and
8) Type of opioid: strong, weak, or other.

Univariate and multivariate analyses were used to describe opioid use patterns at end-of-life, including the average number of prescriptions and the average MEQ dispensed in the year prior to death. The univariate analysis to estimate the number of opioid prescriptions dispensed within each study period used a person-days at risk method for each of the study covariates including sex, age group at diagnosis, cancer type, prognostic tier and urban or rural residence. Person-days at risk takes exposure time into account. Each person’s actual time at-risk is useful for follow-up studies such as ours because exposure time (prognosis) varies by cancer type and other co-variates such as age.

The multivariate analyses were controlled for sex, age group at diagnosis, prognostic tier and urban or rural residence. Cancer type was excluded from the multivariate model because it is highly correlated with prognostic tier.

Univariate regression analyses were used to estimate MEQ consumption for each of the study covariates. Poisson regression analysis was used to model the average number of prescriptions per person per day data, whereas regression analysis was used to model average morphine equivalents per day dispensed per person. Both regression techniques controlled for sex, age group at diagnosis, prognostic tier and urban or rural residence. All univariate and multivariate analyses were conducted using SAS 9.2 (SAS Institute).

## Results

### Demographics and Clinical Characteristics of Study Population

The demographic and clinical characteristics of the end-of-life study population (*n* = 11,498) and the end-of-life study population that was linked to the NSPMP (*n* = 6,186) are shown in Table 1. Since the end-of-life study period included deaths occurring between 1 July 2006 and 31 December 2009, there were only half as many deaths in 2006 compared to the other years under investigation. Males were slightly overrepresented (54%) compared to females (46%). Approximately 80% of the end-of-life study population that was linked to the NSPMP were aged 60 and older at the time of their death. The most commonly occurring cancers accounted for the following percentages of all deaths: lung (24%), colorectal (12%), breast (6%), pancreas (5%) and prostate (4%). Patients most commonly had a low probability of 5-year survival (46%; prognostic tier 3). Nearly two-thirds (64%) resided in urban areas at the time of diagnosis.

**TABLE 1:** Demographic and clinical characteristics of the end-of-life cancer study populations in Nova Scotia from 2005–2009.

Characteristic	Total end-of life study population n (%)^b^	End-of-life study population linked to NSPMP^a^ *n* (%)^c^
Study population	11,498 (100)	6,186 (100)
Year of death		
2006	1,669 (15)	856 (14)
2007	3,331 (29)	1740 (28)
2008	3,307 (29)	1826 (30)
2009	3,191 (28)	1,764 (29)
Sex		
Male	6,222 (54)	3,332 (54)
Female	5,276 (46)	2,854 (46)
Age group at death (years)^d^		
<30	48 (<1)	37 (<1)
30–39	63 (1)	51 (<1)
40–49	351 (3)	279 (5)
50–59	1,043 (9)	803 (13)
60–69	2,132 (19)	1,449 (23)
70–79	3,143 (27)	1742 (28)
80+	4,716 (41)	1824 (29)
Cause of death by cancer site/type		
Oral	112 (1)	87 (1)
Esophagus	210 (2)	142 (2)
Stomach	195 (2)	116 (2)
Colorectal	1,143 (10)	743 (12)
Pancreas	441 (4)	282 (5)
Larynx	51 (<1)	28 (<1)
Lung	2,280 (20)	1,507 (24)
Skin	111 (1)	86 (1)
Breast	506 (4)	363 (6)
Cervix	44 (<1)	33 (<1)
Body of Uterus	100 (1)	53 (<1)
Ovary	176 (2)	104 (2)
Prostate	409 (4)	274 (4)
Bladder	193 (2)	105 (2)
Kidney	204 (2)	136 (2)
Brain	215 (2)	108 (2)
Thyroid	16 (<1)	10 (<1)
Non-Hodgkin’s Lymphoma	313 (3)	171 (3)
Hodgkin’s Lymphoma	14 (<1)	6 (<1)
Leukemia	277 (2)	115 (2)
Liver	65 (1)	40 (<1)
Multiple Myeloma	124 (1)	89 (1)
Other Cancers	1,140 (10)	618 (10)
Non-Cancer Death	3,159 (27)	970 (16)
Prognostic tier (5-year survival percentage)^e^		
Tier 1 (>80%)	1,285 (11)	848 (14)
Tier 2 (50–80%)	2,510 (22)	1,539 (25)
Tier 3 (<50%)	4,544 (40)	2,829 (46)
Other	3,159 (27)	970 (16)
Region at diagnosis^d^		
Urban	7,291 (63)	3,968 (64)
Rural	4,069 (35)	2,167 (35)

a NSPMP, Nova Scotia Prescription Monitoring Program.

b Percentage of total end-of-life study population with each characteristic.

c Percentage of end-of-life study population linked to the NSPMP, with each characteristic.

d Missing values; numbers may not add up to total study population.

e Ellison L and Wilkins K. An update on cancer survival. 2010 [cited 11 November 2018]. Statistics Canada, Catalogue no. 82–003-XPE. Health Reports: 21 (3). Available from: https://www150.statcan.gc.ca/n1/en/pub/82-003-x/2010003/article/11334-eng.pdf?st=X1Jalqn3.

The year of diagnosis and time between diagnosis and death are shown in Supplementary Table S1. Eighty percent of the total end-of-life study population and 85% of the end-of-life study population that was linked to the NSPMP died within 5 years of diagnosis (Supplementary Table S1). For both study populations, the average time between diagnosis and death was 2.7 years with a standard deviation of 4.1 years.

### Type and Characteristics of Opioid Prescriptions

#### Prescriber Type

Eighty-nine percent of prescriptions at end-of-life were written by general practitioners while the remaining 11% were written by specialists (Table 2).

**TABLE 2:** Profile of opioid dispensations dispensed to the end-of-life cancer study population that was linked to the Nova Scotia Prescription Monitoring Program from 2005–2009.

	*n* (%)
Total opioid prescriptions	41,222 (100)
Prescriber Type	
General Practitioner	36,542 (89)
Specialist	4,680 (11)
Opioid Formulation	
Immediate-release	27,316 (66)
Tablets or Capsules	22,101 (54)
Parenteral formulations	3,650 (9)
Oral Solutions	1,474 (4)
Syrups	1,456 (4)
Elixirs	18 (<1)
Powders	86 (<1)
Suppositories	5 (<1)
Modified-release	13,747 (33)
Tablets or Capsules	11,088 (27)
Patches	2,516 (6)
Discs	143 (<1)
Miscellaneous^a^	159 (<1)
Type of Opioid	
Strong Opioids	33,193 (81)
Hydromorphone	20,927 (51)
Morphine	7,956 (19)
Fentanyl	2,659 (6)
Oxycodone	1,651 (4)
Weak Opioids	6,855 (17)
Acetaminophen/Opioid Combination	5,953 (14)
Acetylsalicylic Acid/Opioid Combination	9 (<1)
Codeine	893 (2)
Other	1,174 (3)
Methadone	823 (2)
Meperidine	241 (1)
Pentazocine	67 (<1)
Dextropropoxyphene	43 (<1)
Buprenorphine	0 (0)

a Compounds.

#### Drug Type and Formulation

The formulations and types of opioids in the NSPMP that were linked to the end-of-life study population are found in Table 2. Approximately 81% of all dispensed opioids were strong opioids and 17% were weak opioids (almost all acetaminophen-opioid combinations). Hydromorphone was the most commonly dispensed opioid (51% of all prescriptions) while morphine was the second most commonly dispensed opioid (19% of all prescriptions). Six percent of all prescriptions were for fentanyl while 4% were for oxycodone and 2% were for codeine. Less than 1% of prescriptions were for opioids that are not recommended for cancer pain (i.e., meperidine, pentazocine and dextropropoxyphene). While 2% of prescriptions were for methadone, it is not known if these were for cancer-related pain or other use (e.g., daily prescriptions for opioid maintenance therapy for dependence). Sixty-six percent of all opioid prescriptions were for immediate-release formulations and 33% were for modified-release products.

#### Annual Rate of Prescriptions

There was an increasing trend from 2006 to 2009 related to the number of prescriptions received per patient per year in the total end-of-life study population (Table 3). In 2006, the adjusted mean number of prescriptions per person per year was 5.9 while in 2009 it was 7.0 (*p* < 0.0001). The mean annual rate of prescriptions did not differ by sex. Older patients (age 80 and over) received the fewest prescriptions (mean 3.9/year) while those age 40–49 received the most (mean 14.5/year). Cancer type had an effect on the number of prescriptions dispensed per year: patients with pancreatic (mean 9.7/year), lung (mean 7.7/year), prostate (mean 7.1/year) or oral (mean 7.0/year) cancer received the greatest mean number of prescriptions per year (*p*<0.0001). Patients in prognostic tier 1 had fewer prescriptions (mean 5.7/year) than those in prognostic tier 2 (mean 6.0/year) or prognostic tier 3 (mean 7.2/year) (*p* < 0.0001). Urban patients received a greater mean number of prescriptions per year (mean 6.3/year) than rural patients (mean 6.2/year) (*p* < 0.05).

**TABLE 3:** Adjusted average rate of opioid prescriptions^a^ dispensed among the Nova Scotia end-of-life cancer study population from 2005–2009 by selected demographic and clinical characteristics (*n* = 11,498).

Characteristic	*n*	Adjusted average number of prescriptions per person per year	95% CI	Multivariate Poisson regression results (*p*-value)
Study population	11,498	—		
Year of death				*p* < 0.0001
2006	1,669	5.9	(5.7–6.2)	
2007	3,331	6.3	(6.1–6.5)	
2008	3,307	6.7	(6.4–6.9)	
2009	3,191	7.0	(6.8–7.3)	
Sex				*p* = 0.617
Male	6,222	6.3	(6.1–6.5)	
Female	5,276	6.3	(6.1–6.5)	
Age group at death (years)^b^				*p* < 0.0001
<30	48	11.4	(10.1–12.8)	
30–39	63	10.3	(9.3–11.3)	
40–49	351	14.5	(13.8–15.1)	
50–59	1,043	11.7	(11.3–12.1)	
60–69	2,132	9.0	(8.7–9.3)	
70–79	3,143	6.3	(6.1–6.5)	
80+	4,716	3.9	(3.7–4.0)	
Cause of death by cancer site/type				*p* < 0.0001
Oral	112	7.0	(6.5–7.5)	
Esophagus	210	6.0	(5.6–6.4)	
Stomach	195	6.7	(6.2–7.2)	
Colorectal	1,143	6.3	(6.1–6.5)	
Pancreas	441	9.7	(9.2–10.2)	
Larynx	51	4.7	(4.1–5.4)	
Lung	2,280	7.7	(7.5–7.9)	
Skin	111	4.2	(3.8–4.6)	
Breast	506	4.9	(4.7–5.2)	
Cervix	44	6.2	(5.5–6.9)	
Body of Uterus	100	4.3	(3.9–4.7)	
Ovary	176	3.9	(3.5–4.2)	
Prostate	409	7.1	(6.8–7.4)	
Bladder	193	5.7	(5.3–6.2)	
Kidney	204	6.3	(5.9–6.7)	
Brain	215	2.5	(2.3–2.8)	
Thyroid	16	6.5	(5.2–8.2)	
Non-Hodgkin’s Lymphoma	313	4.0	(3.8–4.3)	
Hodgkin’s Lymphoma	14	3.9	(2.8–5.3)	
Leukemia	277	2.2	(2.0–2.4)	
Liver	65	3.9	(3.4–4.5)	
Multiple Myeloma	124	6.1	(5.6–6.6)	
Other Cancers	1,140	6.7	(6.4–6.9)	
Non-Cancer Death	3,159	1.9	(1.8–2.0)	
Prognostic tier (5-year survival percentage)^b^				*p* < 0.0001
Tier 1 (>80%)	1,285	5.7	(5.5–5.8)	
Tier 2 (50–80%)	2,510	6.0	(5.8–6.1)	
Tier 3 (<50%)	4,544	7.2	(7.1–7.4)	
Other	3,159	1.9	(1.9–2.0)	
Region at diagnosisc				*p* < 0.05
Urban	7,291	6.3	(6.1–6.5)	
Rural	4,069	6.2	(5.9–6.4)	

a As estimated from the multivariate model containing all of the above explanatory factors.

b Ellison L and Wilkins K. An update on cancer survival. 2010 [cited 11 November 2018]. Statistics Canada, Catalogue no. 82–003-XPE. Health Reports:21 (3). Available from: https://www150.statcan.gc.ca/n1/en/pub/82-003-x/2010003/article/11334-eng.pdf?st=X1Jalqn3.

c Missing values; numbers may not add up to total study population.

#### Average Morphine Equivalents per Day

The average MEQ for the end-of-life population that was linked to the NSPMP are noted in Table 4. There was no difference in adjusted average MEQ from 2006–2009. Males received a higher mean daily dose (27.3 MEQ) compared to females (24.9 MEQ) (*p* < 0.01). Patients aged 80 years and older received lower doses than all other age groups (17.0 MEQ), with the largest dose received by patients aged 40–49 (80.2 MEQ) (*p* < 0.0001). The MEQ varied significantly by cancer type with pancreatic (33.4 MEQ), prostate (33.3 MEQ) and multiple myeloma (31.0 MEQ) receiving the most MEQ (*p* < 0.0001). Patients in prognostic tier 1 received more MEQ (27.1) than those in prognostic tier 2 (26.0) or prognostic tier 3 (26.2). There was no dosage difference in urban versus rural residence at diagnosis.

**TABLE 4:** Adjusted average morphine equivalents per day (MEQ) dispensed per person among the Nova Scotia end-of-life cancer study population by selected demographic and clinical characteristics from 2005–2009 (*n* = 6,148).

Characteristic	*n*	Adjusted mean MEQ^a^ ^,^ ^b^ ^,^ ^c^	95% CI	Multivariate regression results (*p*-value)
Study population	6,148			
Year of death				*p* = 0.12
2006	847	28.0	(25.0–31.3)	
2007	1,731	27.3	(27.3–30.3)	
2008	1,818	26.7	(24.2–29.6)	
2009	1,752	26.1	(23.5–29.1)	
Sex				*p* < 0.01
Male	3,310	27.3	(24.7–30.3)	
Female	2,838	24.9	(22.4–27.7)	
Age group at death (years)^d^				*p* < 0.0001
<30	37	53.9	(36.5–79.6)	
30–39	51	62.9	(45.1–87.6)	
40–49	278	80.2	(68.2–94.4)	
50–59	801	57.0	(50.6–64.2)	
60–69	1,441	41.8	(37.6–46.4)	
70–79	1730	27.3	(24.7–30.3)	
80+	1809	17.0	(15.2–18.9)	
Cause of death by cancer site/type				*p* < 0.0001
Oral	87	19.4	(15.1–24.9)	
Esophagus	141	21.3	(17.4–25.9)	
Stomach	115	23.0	(18.5–28.6)	
Colorectal	738	27.3	(24.7–30.3)	
Pancreas	282	33.4	(28.8–38.7)	
Larynx	28	24.8	(16.1–38.0)	
Lung	1,502	26.2	(24.1–28.4)	
Skin	86	25.9	(20.1–33.4)	
Breast	361	22.4	(19.4–26.0)	
Cervix	33	24.2	(16.2–36.3)	
Body of Uterus	53	23.8	(17.3–32.8)	
Ovary	104	21.2	(16.7–26.8)	
Prostate	271	33.3	(28.8–38.6)	
Bladder	104	27.2	(21.7–34.1)	
Kidney	134	26.9	(21.9–33.0)	
Brain	107	12.8	(10.2–16.1)	
Thyroid	10	25.7	(12.6–52.4)	
Non-Hodgkin’s Lymphoma	170	19.9	(16.6–24.0)	
Hodgkin’s Lymphoma	6	16.0	(6.3–40.2)	
Leukemia	114	16.9	(13.5–21.0)	
Liver	40	20.5	(14.3–29.3)	
Multiple Myeloma	89	31.0	(24.2–39.6)	
Other Cancers	614	27.0	(24.2–30.1)	
Non-Cancer Death	959	14.0	(12.7–15.4)	
Prognostic tier (5-year survival percentage)^e^				*p* < 0.0001
Tier 1 (>80%)	843	27.1	(24.4–30.0)	
Tier 2 (50–80%)	1,528	26.0	(23.9–28.3)	
Tier 3 (<50%)	2,818	26.2	(24.4–28.2)	
Other	959	14.2	(12.9–15.7)	
Region at diagnosis				*p* = 0.09
Urban	3,950	27.3	(24.7–30.3)	
Rural	2,147	28.8	(25.9–32.1)	

a MEQ, morphine equivalents per day.

b Parenteral opioids were inadvertently grouped with oral liquids and dosages divided by 5 due to an error in data analysis; however, since parenteral opioids made up 9% of all opioid prescriptions dispensed, we expect this to have minimal effect on the data.

c MEQ, used for oxycodone (0.334) and codeine (0.05) were lower than the current standard based on local consideration at the time of the study that these were weaker opioids. These two opioids made up approximately 6% of the total study opioids.

d Missing values; numbers may not add up to total study population.

e Ellison L and Wilkins K. An update on cancer survival. 2010 [cited 11 Nov 2018]. Statistics Canada, Catalogue no. 82-003-XPE. Health Reports:21(3). Available from: https://www150.statcan.gc.ca/n1/en/pub/82-003-x/2010003/article/11334-eng.pdf?st=X1Jalqn3.

## Discussion

Our study examined opioid analgesic use at end-of-life in patients with cancer for a large real-world population in Nova Scotia and determined factors, trends and patterns associated with type and dose of opioid prescribed. There is limited randomized controlled evidence to determine opioid management at end-of-life and our study provides information regarding how prescribers provide opioid therapy in the face of uncertain evidence (Kumar, 2011).

Similar to others, our study found fewer prescriptions per year and lower daily MEQ for older patients. In addition, older patients at end-of-life were less likely to be linked to the NSPMP (i.e., less likely to receive opioid prescriptions): only 39% of patients aged 80 years or older were linked to the NSPMP compared with 64% of patients under age 80. We were unable to determine reasons for this. Patients with cognitive impairment may have difficulty in communicating pain (Ripamonti et al., 2011) and physicians may be reluctant to give higher doses of morphine in older individuals with multiple morbidities. Older patients may also be more likely to be admitted to hospital for end-of-life care and receive opioids in that setting. It is also possible that older patients were more likely to have died due to a cause other than cancer, for which they may not have required opioids. For example, in Canada, cancer and heart disease accounted for approximately half of the deaths in people age 65 and over, and heart disease outranked cancer as the cause of death in people age 85 and older in 2012 (Statistics Canada, 2012).

Our study demonstrated that the majority of encrypted patient identifications linked to the NSPMP at end-of-life were prescribed strong opioids. Opioids that were not recommended in guidelines were rarely used. Some jurisdictions report that physicians are reluctant to use strong opioids (Gao et al., 2014). A Danish study reported only 40% of opioids used by patients with cancer were strong opioids in 1994–1998 (Jarlbaek et al., 2005). Between 2005–2012 in the United Kingdom, 48% of patients with cancer were prescribed a strong opioid in the last year of life (Ziegler et al., 2016). More recently, in Australia, initiation of a strong opioid occurred in 55.8% of patients with cancer and 28.2% of those without cancer between 2013 and 2017 (Lalic et al., 2019). In France in 2012–2016, patients with metastatic bone cancer were found to have an increased dose of strong opioids prescribed once their situation was deemed palliative (Tarot et al., 2021). Our study found that most strong opioid prescriptions were for hydromorphone followed by morphine. In a population-based Ontario study of clinical indications for initiation of opioid therapy, hydromorphone was most commonly prescribed among individuals initiating opioids for cancer or palliative care in 2015–2016 (Pasricha et al., 2018).

Fentanyl patches tend to be used in patients with intolerable morphine side effects or lack of ability to use the oral route, but they are more expensive and have exception criteria for reimbursement on the Nova Scotia Formulary. Fentanyl was used in only 6% of patients in our study. This contrasts with a study of patients with cancer in Taiwan where fentanyl was the opioid prescribed most commonly in 2007 (288 defined daily dose for statistical purposes per million inhabitants per day (S-DDD)), followed by morphine (135 S-DDD) and then codeine (37 S-DDD) (Pan et al., 2013). However, hydromorphone and oxycodone are not available in Taiwan (Pan et al., 2013). In Denmark, fentanyl was used in 11% of patients with cancer in 1998 (Jarlbaek et al., 2005).

Oxycodone was used in 4% of patients. Only 2% of patients received codeine, which is metabolized to morphine in the liver. Codeine may have adverse effects in patients with the CYP2D6 ultrarapid metabolizer phenotype, may be ineffective in poor CYP2D6 metabolizers, and is subject to many drug interactions (Leppert, 2011; Fallon et al., 2018). There was also less than 1% use of meperidine, pentazocine, or dextropropoxyphene which are not recommended as first line drugs.

Our study found that immediate-release formulations of opioids, which are often used for breakthrough pain, were commonly prescribed. Use of immediate-release formulations of opioids is supported by several different recommendations (Global Year against cancer pain 2008-2009, 2015; Potter, 2006).

The average number of opioid prescriptions dispensed to the total end-of-life study population (*n* = 11,498) increased over the study period from 5.9 to 7.0 (*p* < 0.0001). However, since only 53.8% of the total end-of-life population was linked to the NSPMP (and therefore dispensed opioids), we would expect the average number of opioid prescriptions per person per year to be approximately 11–13 for the linked end-of-life population. A study of a health maintenance organization in Israel where patients with cancer receive opioids free of charge noted that these patients received a mean of 5.6 prescriptions per year in 2006 (Shvartzman et al., 2009).

The average MEQ dispensed per patient per year in the end-of-life study population that was linked to the NSPMP (*n* = 6,148) was between 26.1 and 28.0. When these numbers are doubled to calculate oral morphine equivalents, the numbers remain lower than that reported in a study from Israel that found the oral morphine equivalents per day per cancer patient was 113.8 in 2006 (Shvartzman et al., 2009). Similarly, a US study of patients in the 30 days prior to death or hospice enrollment reported higher morphine doses: 85.6 oral morphine milligram equivalents per day in 2007 (Enzinger et al., 2021). However, our results are higher than those found in a Danish study where 10.7 g of oral morphine equivalents per cancer patient per year were used in 1998; this translates to approximately 29.3 MEQ per patient per year (Jarlbaek et al., 2005).

In the wake of the opioid crisis, it is important to reinforce that opioids remain the mainstay of therapy to treat pain in patients with cancer. As health care professional regulatory bodies work to ensure safe opioid prescribing for the broader population (Donroe et al., 2018), physicians may become more reluctant to prescribe opioids and it is possible that there may be unintended consequences of reduced opioid prescribing. This may lead to suboptimal pain management in patients with cancer including those at end-of-life. An Ontario study noted that trends in opioid prescribing may affect patients with or without cancer similarly (Barbera et al., 2018). In spite of the implementation of a provincial symptom screening program with a goal of improving symptom management in patients with cancer, opioid prescription rates did not change in elderly patients with cancer (Barbera et al., 2017). In a younger population, the annual proportion of patients with an opioid prescription decreased from 2004 to 2013 for both cancer and noncancer patients (Barbera et al., 2018). In a US study of Medicare patients with cancer, opioid use in the 30 days prior to death or hospice enrollment declined from 2007 to 2017, while pain-related emergency room visits increased, suggesting that pain control at end-of-life may be worsening in patients with cancer (Enzinger et al., 2021). Similarly, in a US study of patients with solid tumour cancers in the 30 days prior to death, opioid use also declined from 44.7% in 2007–2009 to 26.7% in 2013–2015 (McDermott et al., 2017). One explanation for these findings may be that policies aiming to prevent misuse of opioids have unintentionally led to reduced access to opioids in patients at end-of-life. This may not be limited to patients with cancer. Furuno et al. (2021) found that the frequency of opioid prescribing decreased from 2010 to 2018 in patients being discharged from hospital to hospice care.

A United Kingdom study demonstrated that patients with cancer who receive palliative care were more than twice as likely to receive a strong opioid in the last year of life compared to those who were not provided with palliative care in 2010–2012 (Ziegler et al., 2018). The provision of palliative care in China was also associated with increased prescriptions for strong opioids (Lam et al., 2021). This highlights the role of the palliative care team in ensuring appropriate access to opioids at the end-of-life. However, patients who had not received an opioid prescription were less likely to receive palliative care (Craigs et al., 2018) and in many jurisdictions, access to opioids and palliative care services is limited (Herce et al., 2014; Nambiar et al., 2021; Ngoma et al., 2021).

There may also be evidence of racial inequities in the timing of access to opioids in patients approaching end-of-life (Gurney et al., 2021). In a New Zealand study from 2007 to 2016, 74% of all patients with advanced lung cancer accessed strong opioids within 12 months of diagnosis; however, Maori patients were more likely to first access strong opioids in the 2 weeks prior to death than non-Maori patients (Gurney et al., 2021). These findings highlight that in addition to being cognizant of the special circumstances surrounding opioid prescribing for patients with cancer at end-of-life, prescribers also need to take steps to ensure equitable access to opioids among racialized groups.

### Strengths

Our study was a population-based study using a long-established Cancer Registry (active since 1964) linked to the Nova Scotia Prescription Monitoring Program (active since 1992) in which the vast majority of opioids are legislated to be reported. This is the first time these two databases have been linked. We present longitudinal data for both patients with cancer and patients receiving opioids in a province with a longstanding prescription monitoring program. This adds to the evidence related to opioid use by patients with cancer in jurisdictions with a prescription monitoring program (Haffajee et al., 2015; Sproule, 2015; Finley et al., 2017). In addition, we studied an end-of-life cohort. Detailed information on cancer type, prognostic tier and opioid dose, type and route of administration were available. Because we had individual level patient data, we were able to calculate prescribed daily doses (University of Manitoba, 2005) rather than defined daily doses which are a technical unit and reported in some studies when only sales data or pharmacy inventory data are available (Wettermark et al., 2019). We have presented detailed information that can be used for a specific comparison among patients with cancer at end-of-life. In addition, this study provides careful contextual information for other jurisdictions to do a comparison as it is known that the type of opioid and prescribing rate varies by jurisdiction (Jani et al., 2021). This data may also be useful for palliative care practitioners as they face many challenges, such as an increasing role in managing chronic pain in addition to caring for those with advanced illness, as well as managing and treating addiction (Merlin et al., 2019).

### Limitations

The study period was 2005–2009; therefore, opioid prescribing trends may have changed over time. In particular, opioid prescribing may have decreased since this study was conducted as national and regulatory bodies have taken measures to respond to the opioid crisis. For example, a Special Advisory Committee on the Epidemic of Opioid Overdoses was formed in Canada in 2016 in response to the growing opioid crisis (Pan-Canadian Public Health Network, 2021). However, these data can serve as a baseline against which changes in prescribing can be analyzed over time, particularly for patients with cancer in the last year of life.

Opioid use was limited to prescriptions dispensed in community pharmacies within Nova Scotia and did not include any prescriptions dispensed outside the province nor opioids used within hospital settings. Some of the cancer cases that were not linked to the NSPMP may have been in hospital or a palliative care unit which may impact the type and amount of opioid prescribed (Howell et al., 2011).

We were not able to determine whether patients in our study were under-treated with opioids as we did not measure pain control; however, the lower MEQ reported here compared to some other jurisdictions would support this idea. We were unable to determine side effects of the opioids including central nervous system (sedation, confusion, cognitive impairment), gastrointestinal (constipation, nausea, vomiting diarrhea) or other side effects which may have affected the choice of agent (Suh et al., 2004; Wilsey et al., 2010). We were also unable to determine specific physician factors influencing the variation in prescribing including physician characteristics, beliefs and knowledge of pain management for cancer patients at end-of-life (Grant et al., 2009; Mazoyer et al., 2017). We did not study other barriers to opioid prescription such as patient and family attitudes (Jacobsen et al., 2014; Mazoyer et al., 2017; Wright et al., 2019).

We did not look at health service factors changing over time such as the availability of palliative care services or the co-prescription of other treatment modalities such as psychological interventions and physiotherapy. We also did not look at changes in cancer incident rates that may have occurred over the course of this study. The inclusion of non-cancer deaths is a limitation of this study as is the assumption that patients used opioids to treat cancer pain. However, death due to cancer was reported for 84% of patients in this study. Multiple causes of death, and the lack of clinical information to determine whether end-of-life treatment strategies were used, are common limitations that are not specific to studies of patients with cancer (Gershon et al., 2018).

We did not measure concurrent use of multiple opioids, which is sometimes used (e.g., morphine plus hydromorphone) (Lauretti et al., 2003; Gao et al., 2014). We were not able to measure tramadol utilization as tramadol was not included in the NSPMP at the time of this study. Codeine in combination with acetaminophen or acetylsalicylic acid is available without a prescription when the codeine component is 8 mg or less per tablet; therefore, these combination tablets are not monitored under the NSPMP. We did not look at concomitant use of opioids with other adjuvant analgesics (Leppert, 2011). Of note, equianalgesic dose recommendations for the calculation of MEQ have changed over time (Pain and symptom management, 2017; Fallon et al., 2018; Pharmacist’s letter, 2012; Government of Canada, 2009).

### Future Considerations

Our findings can be used to increase awareness of factors associated with type and dose of opioid prescribed among general practitioners, and to inform opioid standards for professional regulatory bodies to improve pain management in patients with cancer at end-of-life. Future study is needed to assess adherence to standards by general practitioners. Since general practitioners were identified as the primary prescriber of opioids in this population, they should be prioritized for educational programs, audit and feedback, and clinical decision support tools. Our approach can also be used as health systems change their opioid policies and educational interventions. For example, in response to the COVID-19 pandemic, Health Canada issued temporary exemptions under the Controlled Drugs and Substances Act to allow pharmacists to provide continuity of care to vulnerable populations in recognition of the importance of treating chronic pain (Health Canada, 2020). Pharmacist prescribing is one example of an innovative strategy that may be used to improve access to opioids for patients with chronic pain, including those with cancer at end-of-life. Further work is needed to determine the impact of prescribing choices (drug type, route dose, duration, monitoring) on patient pain control and quality of life in patients with cancer at end-of-life. Future study can also address how opioid prescribing has changed over time, and whether efforts to reduce opioid prescribing in response to the opioid crisis have affected patients with cancer at end-of-life in Nova Scotia.

## Data Availability

The datasets presented in this article are not readily available because the policies and procedures established by the data steward of the Nova Scotia provincial government, which govern the disclosure of the information in this study, do not allow public sharing of dis-aggregated data. Requests to access the datasets should be directed to Ingrid Sketris, ingrid.sketris@dal.ca.
