# Supplementary material for: Opioid Use at End-Of-Life Among Nova Scotia Patients With Cancer
Source: Front Pharmacol. 2022 Mar 24;13:836864. doi: 10.3389/fphar.2022.836864 (PMC8987150; doi:10.3389/fphar.2022.836864)
Supplement: Supplementary file 1 [file Table1.docx]

**Supplementary Table 1. Year of diagnosis and time between diagnosis and death for the end-of-life cancer study populations in Nova Scotia.**

| Characteristic | Total end-of life study population  n (%) | End-of-life study population linked to NSPMP^a^  n (%) |
| --- | --- | --- |
| Study population | 11498 (100) | 6186 (100) |
| Year of diagnosis |  |  |
| 1991-1995 | 764 (7) | 282 (5) |
| 1996-2000 | 1098 (10) | 498 (8) |
| 2001-2005 | 2737 (24) | 1628 (26) |
| 2006-2010 | 6899 (60) | 3778 (61) |
| Time between diagnosis and death (years) |  |  |
| < 1 | 5458 (47) | 2755 (45) |
| 1-5 | 3805 (33) | 2466 (40) |
| 6-10 | 1252 (11) | 579 (9) |
| 11-15 | 905 (8) | 357 (6) |
| 16+ | 78 (<1) | 29 (<1) |

^a^NSPMP, Nova Scotia Prescription Monitoring Program
