# Supplementary figures and images for: Opioid Use at End-Of-Life Among Nova Scotia Patients With Cancer
Source: Front Pharmacol. 2022 Mar 24;13:836864. doi: 10.3389/fphar.2022.836864 (PMC8987150; doi:10.3389/fphar.2022.836864)

1    Supplementary Figure. 1. Data linkage.

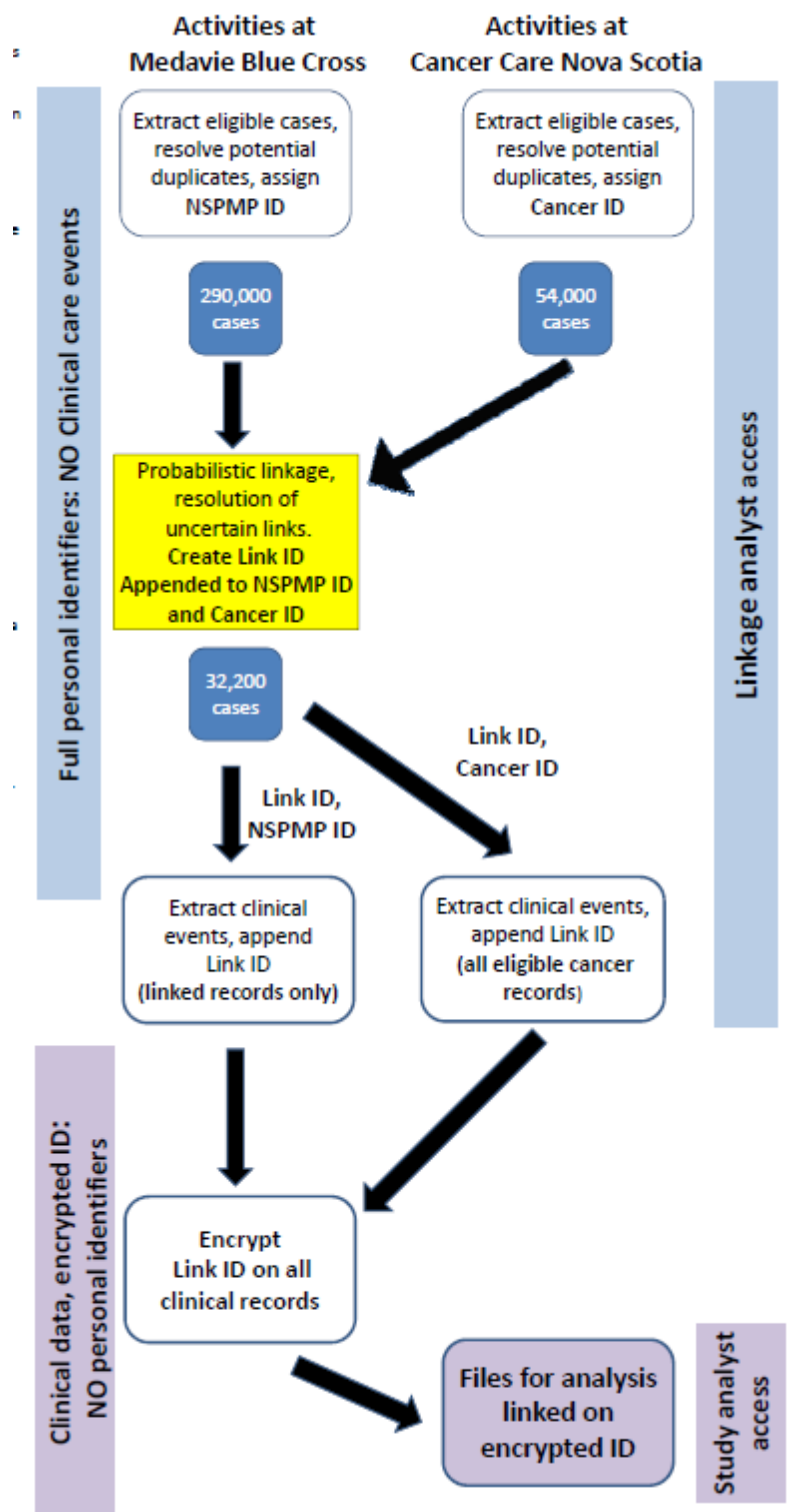

Supplement: Supplementary file 2 [file Image1.pdf]
